# Application of Bayesian statistics for radiation dose assessment in mixed beta-gamma fields

**DOI:** 10.1007/s00411-021-00906-w

**Published:** 2021-04-16

**Authors:** I. Słonecka, J. Krasowska, Z. Baranowska, K. W. Fornalski

**Affiliations:** 1 Central Laboratory for Radiological Protection (CLOR), Konwaliowa 7, 03-194 Warszawa, Poland; 2 Faculty of Physics, Warsaw University of Technology, Koszykowa 75, 00-662 Warszawa, Poland; 3 National Centre for Nuclear Research (NCBJ), Sołtana 7, 05-400 Otwock-Świerk, Poland

**Keywords:** Mixed radiation, Bayesian statistics, Monte Carlo, Dosimetry, Dose assessment

## Abstract

The present paper proposes a novel method, based on Bayesian statistics, as a new approach in the field of thermoluminescence dosimetry for the assessment of personal doses in mixed beta-gamma radiation fields. The method can be utilized in situations when the classical way of dose calculation is insufficient or impossible. The proposed method uses a prior function which can be assigned to the unknown parameter and the likelihood function obtained from an experiment, which together can be transformed into the posterior probability distribution of the sought parameter. Finally, the distribution is converted to the value of the dose. The proposed method is supported by analytical and Monte Carlo calculations, which confirmed the results obtained through the Bayesian approach.

## Introduction

Mixed radiation fields are composed of at least two different types of radiation (e.g. neutron and gamma), different energies (e.g., neutron energy spectrum) or different radiation sources (e.g. ^137^Cs + ^90^Sr). Mixed radiation fields are very common—even one radionuclide can disintegrate in a few possible ways and emit different particles with different energies. Radiation-induced risks from mixed radiation fields concern, for example, nuclear medicine, nuclear power plants, flights, space flights and other places/environments/locations where the possibility of contact with several sources exists.

Generally, individual particles included in a mixed radiation field differ in linear energy transfer along the particle path within the absorbent. As a result, the shape of the particles’ path and range are different. The various microscopic structures of ionization paths and the different ways of interacting with the DNA of cells are the reason that the mixed radiation components show different biological effectivenesses, even at the same level of absorbed dose. Consequently, the biological impact of these components on the human body is also different. In mixed fields there may also exist particles that are different in nature but finally interact with matter in the same way, e.g. beta and gamma radiation. For this reason, the radiation weighting factor was set to *w*_*R*_ = 1 for both. Gamma radiation ionizes matter through interactions of secondary electrons (Compton electrons, photoelectrons, or electrons and positrons pairs). However, what makes them different is their penetration strength, which depends on energy. Direct beta radiation is strongly absorbed from the beginning of its path in matter, while gamma radiation (especially with higher energies) will penetrate much deeper. Therefore, in the case of mixed radiation fields, assessment of the total dose is not enough, and there is a strong need to calculate individual absorbed doses of each component separately. Despite the existing solutions for dosimetry in mixed radiation fields, searching for simplifications and improvements, as well as for new, better methods, is advisable (Delgado 2000).

The biggest problem in assessing doses in mixed radiation fields is the accurate assessment of their composition. To facilitate dose assessment, the radiation type which is considered less dangerous (usually beta radiation) is often neglected. On the other hand, beta radiation may cause significant risk for example for the skin or the lens of the eye. Therefore, assessment of doses from beta radiation is important, and resulting doses have to be compared with worldwide equivalent dose limits: 20 mSv $\cdot$ y^−1^ for the whole body, 500 mSv $\cdot$ y^−1^ for skin and limbs, and 20 mSv $\cdot$ y^−1^for the lens of the eye. In practice, beta-gamma mixed radiation is one of the most often occurring mixed fields (Delgado 2000).

Beta radiation is generally considered to be weakly penetrating (the exception is, for example, beta radiation emitted during the decay of ^16^ N with a high beta endpoint energy of 10.4 meV). In such cases, the operational quantity for individual monitoring is personal dose equivalent, *H*_*p*_*(d)* at *d* = 0.07 mm depth for skin, and at *d* = 3 mm depth for the lens of the eye (*H*_*p*_*(0.07)* and *H*_*p*_*(3)*, respectively). In contrast, for photons, the depth of tissue is considered as *d* = 10 mm, so *H*_*p*_*(10)* is the quantity to be used (ICRU 1998; Endo 2016).

In practice, thermoluminescence dosimeters (TLDs) are the most common detectors for personal dosimetry. TLDs are small and cheap and have a linear dose–response up to about 50 Sv. Their advantage is that they can be used in mixed beta-gamma fields (Chase and Hirning 2008; Ciupek et al. 2014): using different shielding (filters) of TLD pellets provides accurate results in routine dosimetry. Such filters are used to distinguish and often identify between the different components of a mixed radiation field. However, TLDs also have some limitations that, in many cases, make the correct dose assessment in mixed radiation fields difficult. For example, a significant limitation is that the investigated radiation might show energies which were not used in the calibration procedure. Problems also occur in extremity (Brasik et al. 2006; Papierz et al. 2014) and eye lens dosimetry, where usually only one TLD pellet is available (Carinou et al. 2015), and in cases of accidental overexposures, when it is not possible to determine the calibration factors necessary to convert counts to dose.

In such situations, Bayesian statistics as well as the Monte Carlo methods, which were successfully applied in mixed neutron-gamma radiation fields for biological dosimetry (Brame and Groer 2003; Ainsbury et al. 2013a, b; Pacyniak et al. 2014; Słonecka et al. 2019; Powojska et al. 2018), may be useful. The present paper develops the Bayesian method further, supported by Monte Carlo calculations, as an alternative and very appropriate tool supporting mixed beta-gamma TL dosimetry.

## Materials and methods

### Thermoluminescent dosimeters

The thermoluminescent dosimeters used in the present study were MCP-N round pellets with a diameter of 4.5 mm made of LiF:Mg,Cu,P, were placed in dosimeter cards designed by Mirion Technologies. These dosimeter cards held up to 4 pellets. Pellets in positions one and two were shielded with a 1 mm layer of aluminum, while the pellet in the third position was shielded by a 0.5 mm layer of plastic, and that in the fourth position remained uncovered.

The dosimeter cards were compatible with the RADOS RE-2000 Reader used for the present study. The readout consisted of two phases—the “preheat” phase, that took 5 s, and the “heat” phase, that took 15 s. Pellets were read out at a temperature of 250 $^\circ{\rm C}$ in a constant-temperature mode. The absorbed dose was estimated based on a signal (counts) from the “heat” phase of the TL curve, as an integral of all counts. The readout of the TLD dosimeters was done at Central Laboratory for Radiological Protection (CLOR), Poland, which is accredited by the Polish Centre for Accreditation in accordance with the ISO/IEC 17025 (2018) standard.

### Irradiations

Irradiations were carried out at CLOR at two calibration facilities: the gamma calibration facility equipped with a ^137^Cs gamma source, and the beta calibration facility Beta Secondary Standard BSS 2, equipped with a ^90^Sr/^90^Y beta source (named here ^90^Sr for simplicity). Both facilities are accredited by the Polish Centre for Accreditation in accordance with the EN ISO/IEC 17025 (2018) standard. Calibrations and irradiations were carried out on a PMMA phantom in accordance with the requirements of ISO 4037-3 (1999), ISO 6980 (2004,2006a; b) and IEC 61066 (2006).

The gamma irradiations were done at a distance of 2 m from the ^137^Cs source, with a dose rate of 10 mSv $\cdot$ h^−1^. The uncertainty for the irradiations at the gamma calibration facility was 4% (*k* = 2, which means two sigma uncertainty). The beta irradiations were done at a distance of 30 cm from the ^90^Sr source, with a dose rate of 22 mSv $\cdot$ h^−1^. The uncertainty for the irradiations at the beta facility was 5.4% (*k* = 2).

### Classical approach

Assessment of mixed doses from a beta-gamma radiation field in TL dosimetry is based on the difference in the number of counts read out from the covered—*N*_*C*_ (1st position in the dosimeter case) and uncovered—*N*_*U*_ (4th position) pellets. Taking into account, for example, beta radiation weakly penetrating through an aluminum filter layer, the covered pellet registers the total radiation from ^137^Cs but only part of the beta rays. The exact fraction depends on the source—for ^90^Sr it is 25%. The uncovered pellet registers the total radiation from both sources. This situation is described in Eqs. 1 and 2:

1 $$N_{C} = N_{\gamma } + 0.25N_{\beta }$$

2 $$N_{U} = N_{\gamma } + N_{\beta }$$

where *N*_*γ*_*, N*_*β*_ are the counts from gamma and beta radiation (from the “heat” phase of the TL curve), respectively.

Dose from gamma, ${D}_{\gamma }$ and from beta, ${D}_{\beta }$ can be calculated from Eqs. 3 and 4:

3 $$D_{\gamma } = N_{\gamma } \cdot k_{\gamma }$$

4 $$D_{\beta } = N_{\beta } \cdot k_{\beta }$$

where *k*_*γ*_*, k*_*β*_ are the calibration factors for counts to dose for gamma and beta radiation, respectively.

By entering the *θ* parameter, which is given by Eq. 5, the ratio of counts from the pellets can be calculated as:

5 $$\theta = \frac{{N_{\beta } }}{{N_{\beta } + N_{\gamma } }} = \frac{{N_{\beta } }}{{N_{U} }}$$

where *θ* parameter is from the range [0, 1].

Taking Eqs. 1, 2 and 5, the number of counts from gamma and beta radiation can be written as:

6 $$N_{\gamma } = N_{U} \left( {1 - \theta } \right)$$

7 $$N_{\beta } = N_{U} \cdot \theta$$

With Eqs. 6 and 7, ${D}_{\gamma }$ and ${D}_{\beta }$ can be easily calculated as:

8 $$D_{\gamma } = N_{U} \left( {1 - \theta } \right) \cdot k_{\gamma }$$

9 $$D_{\beta } = N_{U} \cdot \theta { } \cdot k_{\beta }$$

In a classic situation, when there are known sources with known calibration factors, *k*_*x*_ (where *x* = *{β, γ}*) and when the ratio of counts, *θ*, is easy to determine, there is no need for Bayesian or Monte Carlo methods, which require knowledge of complex mathematical operations. However, such cases were initially considered by the authors as means of verifying the correctness of the Bayesian approach in the field of mixed radiation doses, assessing the possibility of these methods as supporting tools and validating the methods.

### Bayesian approach

If some parameters of Eqs. 8 and 9 are difficult or impossible to determine, it is not possible to use the classical approach. In such a case, the Bayesian experimental method can be utilized. The Bayesian approach uses a prior function which is usually expressed in the form of a probability distribution function. The prior function is used to estimate the most probable values of unknown parameters, for example, a *p(*$\theta$*)* prior for the ratio of counts $\theta$, or *p(k*_*x*_*)* priors for the calibration factors *k*_*x*_. Apart from the prior function, Bayesian statistics also includes data obtained in an experiment, named formally as the likelihood function (*L*), which can also have the form of the probability distribution function.

### Likelihood functions

**Fig. 1:**
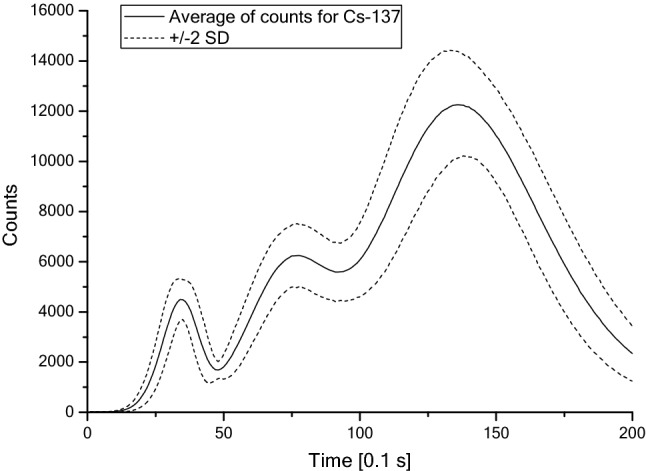
Averaged TL curve for ^137^Cs after exposure to 1 mSv

**Fig. 2:**
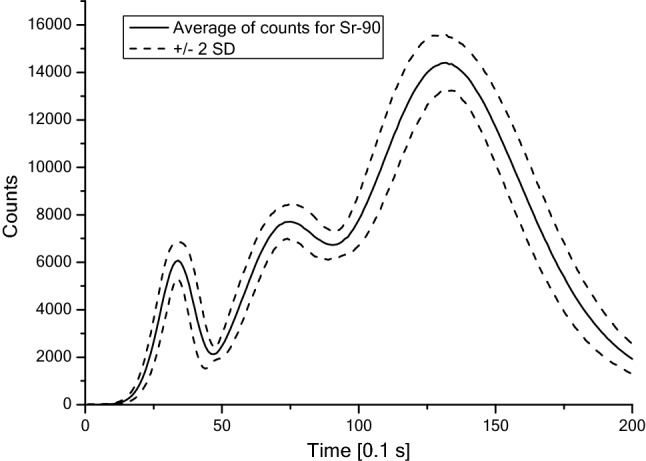
Averaged TL curve for ^90^Sr after exposure to 1 mSv

The likelihood function was obtained here as a response of 150 pellets placed outside the dosimeter cards (without any filter effect) to doses of 1 mSv, separately from ^137^Cs and ^90^Sr. Despite the same irradiation conditions and doses, the responses differ among pellets, due to their individual, slightly different sensitivity. The thermoluminescent curves obtained after readout with a RADOS RE-2000 Reader, averaged over the 150 pellets (solid lines in the Figs. 1, 2) with dispersions expressed as two standard deviations of the mean (dotted lines in the Figs. 1, 2), are shown in Figs. 1 and 2.

**Fig. 3:**
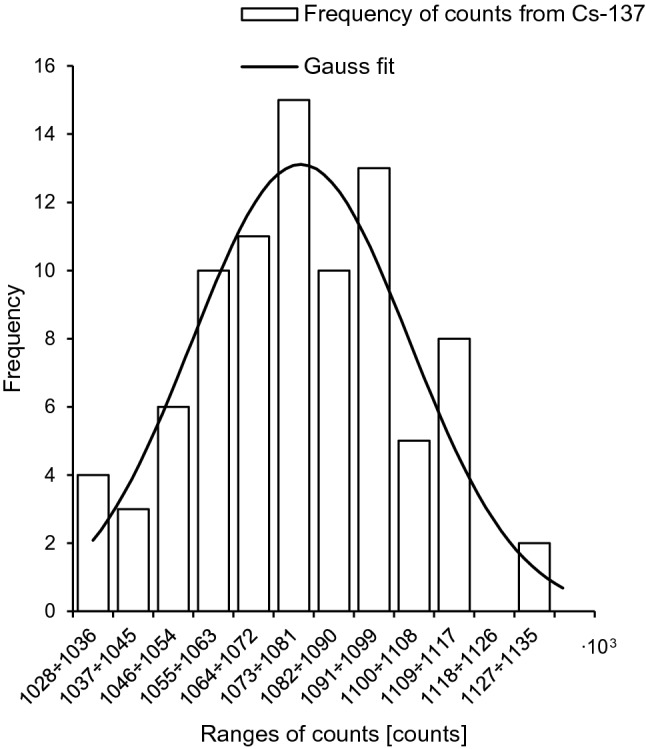
Frequency distribution of counts for ^137^Cs after exposure to 1 mSv. Solid line: Gaussian fit with *R*^2^ = 0.88

**Fig. 4:**
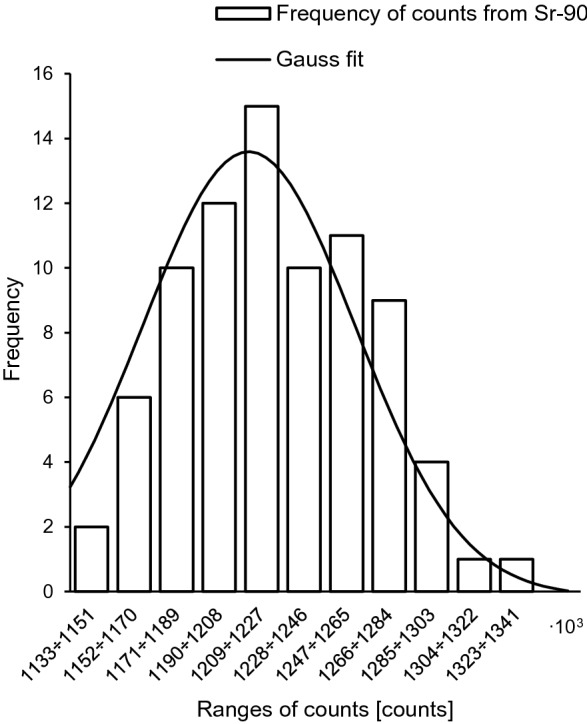
Frequency distribution of counts for ^90^S after exposure to 1 mSv. Solid line: Gaussian fit with *R*^2^ = 0.96

The frequency distributions of the measured counts (taken from the “heat” phase of the TL curves) for the ^137^Cs and ^90^Sr sources take the form of normal distribution (Figs. 3, 4) and are expressed in Eq. 10 for gamma radiation and in Eq. 11 for beta radiation:

10 $$L_{\gamma } (N_{\gamma } ) = \frac{1}{{\sigma_{{N_{U} }} \sqrt {2\pi } }}{\text{exp}}\left( {\frac{{ - \left( {N_{U} - \frac{{D_{\gamma } }}{{\left( {1 - \theta } \right)k_{\gamma } }}} \right)^{2} }}{{2\sigma_{{N_{U} }}^{2} }}} \right)$$

11 $$L_{\beta } (N_{\beta } ) = \frac{1}{{\sigma_{{N_{U} }} \sqrt {2\pi } }}{\text{exp}}\left( {\frac{{ - \left( {N_{U} - \frac{{D_{\beta } }}{{\theta \cdot k_{\beta } }}} \right)^{2} }}{{2\sigma_{{N_{U} }}^{2} }}} \right)$$

### Prior functions

#### a) θ parameter

Potential candidates for the *θ* parameter prior function (also named “prior probability distribution” here) are discussed in detail in (Słonecka et al. 2019). Here, the assumed Gaussian distribution (Eq. 12) has been used only with a precisely known value of the *θ* parameter (Eq. 5), to validate the methodology:

12 $$p\left( \theta \right) = \frac{1}{{\sigma_{\theta } \sqrt {2\pi } }}{\text{exp}}\left( {\frac{{ - (\theta - \hat{\theta })^{2} }}{{2\sigma_{\theta }^{2} }}} \right)$$

#### *b) k*_*x*_* calibration factors*

**Fig. 5:**
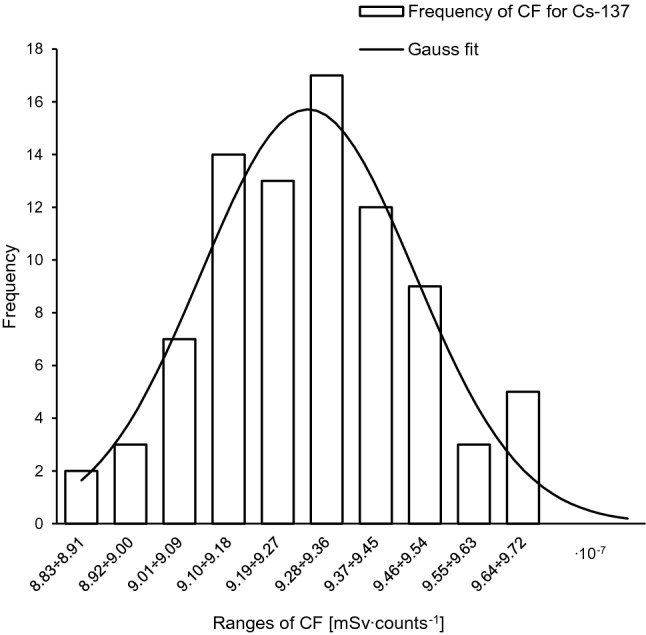
Frequency distribution of calibration factor (CF) for ^137^Cs. Solid line: Gaussian fit with *R*^2^ = 0.87

**Fig. 6:**
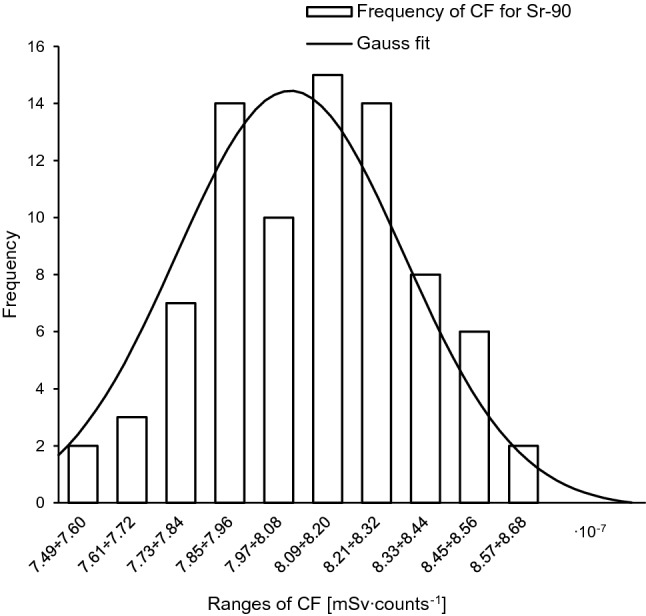
Frequency distribution of calibration factor (CF) for ^90^Sr. Solid line: Gaussian fit with *R*^2^ = 0.94

The calibration factors’ (CF) distributions were obtained experimentally. Pellets outside the dosimeter cards were exposed to 1 mSv for both ^137^Cs and ^90^Sr. The CF frequency distributions have been described also by a Gaussian distribution (Eq. 13) and are presented in Figs. 5 and 6:

13 $$p\left( {k_{x} } \right) = \frac{1}{{\sigma_{{k_{x} }} \sqrt {2\pi } }}{\text{exp}}\left( {\frac{{ - (k_{x} - \widehat{{k_{x} }})^{2} }}{{2\sigma_{{k_{x} }}^{2} }}} \right)$$

### Posterior probability distributions

Consideration of the prior function and the likelihood function is the basis of the Bayesian approach and allows to transform the prior probability distribution into a posterior probability distribution (also named “posterior” here) according to:

14 $$Posterior\,probability\,distribution \propto Likelihood\,function \cdot Prior\,function$$

Equation 14 can be presented in a more detailed form after verification of the considered situation. Let us take some possible options: (1) calibration factors *k*_*x*_ are known, *θ* is unknown, (2) *θ* is known, calibration factors *k*_*x*_ (or one of them) are unknown, (3) *θ* is unknown, calibration factors *k*_*x*_ (or one of them) are unknown. Unknown parameters are typically described in the form of probability distributions. To validate the methods, the expected values and the uncertainties were used in the calculations. Mathematical representations of these situations and the posterior probability distributions are presented in Table 1.

**Table 1:** Considered situations in mixed doses calculations

Option	*θ*	*k*_*x*_	Posterior
1	*p(θ)*	*k*_*x*_ = *const*	$P\left({D}_{x}\right)\propto \int L\left({D}_{x}|\theta \right) p\left(\theta \right)d\theta$
2	*θ* = *const*	*p(k*_*x*_*)*	$P\left({D}_{x}\right)\propto \int L\left({D}_{x}|{k}_{x}\right) p\left({k}_{x}\right)d{k}_{x}$
3	*p(θ)*	*p(k*_*x*_*)*	$P\left({D}_{x}\right)\propto \int \int L\left({D}_{x}|{k}_{x},\theta \right) p\left({k}_{x}\right)p\left(\theta \right)d{k}_{x}d\theta$

Option 1: calibration factors k_x_ are known, *θ* is unknown; Option 2: *θ* is known, calibration factors *k*_*x*_ (or one of them) are unknown; Option 3: *θ* is unknown, calibration factors *k*_*x*_ (or one of them) are unknown

The posterior probability distribution for the situations considered in Table 1 can be obtained by transforming Eq. 14 into a form convenient for the calculations. The probability distributions of the doses can be expressed as (Eqs. 15–17):

15 $$P\left( {D_{x} } \right) = \int\limits_{0}^{1} {L\left( {D_{x} |\theta } \right)p\left( \theta \right)d\theta }$$

16 $$P\left( {D_{x} } \right) = \mathop \int \limits_{ - \infty }^{\infty } L\left( {D_{x} {|}k_{x} } \right) p\left( {k_{x} } \right)dk_{x}$$

17 $$P\left( {D_{x} } \right) = \mathop \int \limits_{0}^{1} \mathop \int \limits_{ - \infty }^{\infty } L\left( {D_{x} {|}k_{x} ,\theta } \right) p\left( {k_{x} } \right)p\left( \theta \right)dk_{x} d\theta$$

where *x* = {*β*, *γ*}.

Finally, doses ${D}_{x}$ can be estimated from the maximum of the distributions or calculated through the first derivative equation (Eq. 18):

18 $$\frac{{dP\left( {D_{x} } \right)}}{{dD_{x} }} = 0$$

The uncertainties of dose estimations, ${\sigma }_{{D}_{x}}$, can be assessed using the Cramér-Rao theorem (Słonecka et al. 2019):

19 $$\sigma_{{D_{x} }} \ge \frac{1}{{\sqrt {\left| {\frac{{\partial^{2} \ln (P\left( {D_{x} } \right)}}{{\partial D_{x}^{2} }}} \right|} }}$$

where *ln(P)* is the natural logarithm of *P(*${D}_{x}$*)* obtained with the maximal likelihood method.

An advantage of the Bayesian methodology is that uncertainties for all parameters (here: *θ* and *k*_*x*_) can be included in the dose assessment, what makes the outcome more realistic. It should be noted, however, that both likelihood and prior functions are assumed to be Gaussian. Of course this assumption has a strong experimental basis (Figs. 3, 4, 5, 6). But these functions can take a different shape in the case of a low number of experimental data, for example. The acceptance criterion for the Gaussian shape of both probability functions was the coefficient of determination (*R*^2^). A high *R*^2^ between 85 and 100% indicates that the regression fits the real data points well.

### Monte Carlo approach

The methods used in the present study were additionally verified and checked using the Monte Carlo technique. For this, a dedicated computational code written in Phyton was used, where the major input parameter (namely, *θ* and/or *k*) can be randomly selected from the appropriate probability distribution function (Eqs. 12 and 13), respectively. The expected value and the corresponding uncertainty can be taken from the input data in two ways: when *N* and *k*_x_ are given by exact values, or when *N* is given by an exact value and *k*_*x*_ is selected from the appropriate probability distribution function. Finally, *θ* can be calculated with the use of the computational code; the uncertainty of *θ* was calculated using the exact differential method. Afterwards, the randomized parameter is put into Eqs. 10 and 11, and the proper value of *N* is analogically randomly selected using an iterative approach. Finally, Eqs. 8 and 9 were used to calculate both values of doses, ${D}_{x}$. This algorithm was repeated several times (preferably 100,000 iterations) to create the ${D}_{x}$ distribution and to determine its preferable value and corresponding uncertainty.

The applied Monte Carlo method is nothing more than a randomized iterative realisation of the Bayesian method described before. However, it allows calculation of the doses in three different conditions (Table 1) and, what is an additional advantage, it is a useful tool to create virtual numerical data which can be considered as a good approximation of real experiments. In the future, this approach could be used to estimate counts from (virtual) radiation sources that are not available at CLOR, but are used in practice, such as ^14^C or ^99m^Tc, and to develop a basic set of calibration factors.

## Results and discussion

### Calibration factors

Calibration factors were obtained (Table 2) by irradiating pellets with a dose of 1 mSv of gamma and beta radiation and using Eqs. 3 and 4.

**Table 2:** Calibration factors for ^137^Cs and ^90^Sr for covered and uncovered pellet positions

Pellet position	*k*_*γ*_ ± SD* [mSv·counts^−1^]	*k*_*β*_ ± SD* [mSv·counts^−1^]
Covered	9.29·10^–7^ ± 0.20·10^–7^	2.88·10^–6^ ± 0.16·10^–6^
Uncovered		8.17·10^–7^ ± 0.28·10^–7^

^*^SD standard deviation

### Mixed doses assessment

TLD dosimeters were exposed to a mixed beta-gamma radiation field with different fractions from ^90^Sr and ^137^Cs (column 1 in Table 3). In each case, total doses were about 1 mSv total. The results of counts for covered (C) and uncovered (U) pellets are reported in Table 3. Counts were reduced by background values, doses were calculated in accordance with Eqs. 3 and 4, and the calibration factors were taken from Table 2. $\stackrel{-}{{N}_{\beta }},\stackrel{-}{{N}_{\gamma }}$ were calculated following Eqs. 1 and 2. The results for irradiation doses (reference doses), as well as those from the classical approach, are also presented in Table 3.

**Table 3:** Results of mixed field exposure for physical dosimetry

% *β* + % *γ*	Pellet position	$\overline{N}$ [counts]	$\stackrel{-}{{N}_{\beta }}$[counts]*	$\stackrel{-}{{N}_{\gamma }}$[counts]*	Reference dose		Classical approach **	
					${D}_{\beta }[mSv]$	${D}_{\gamma }[mSv]$	${D}_{\beta }[mSv]$	${D}_{\gamma }[mSv]$
10% *β* + 90% *γ*	C	993,801	148,736	972,883	0.120 ± 0.007	0.900 ± 0.036	0.122 ± 0.021	0.904 ± 0.154
	U	1121,619						
30% *β* + 70% *γ*	C	856,239	410,927	769,773	0.313 ± 0.017	0.700 ± 0.028	0.336 ± 0.057	0.715 ± 0.122
	U	1180,700						
50% *β* + 50% *γ*	C	695,997	646,995	550,514	0.511 ± 0.028	0.500 ± 0.020	0.529 ± 0.090	0.511 ± 0.087
	U	1197,509						
70% *β* + 30% *γ*	C	605,507	944,498	369,382	0.717 ± 0.039	0.300 ± 0.012	0.772 ± 0.131	0.343 ± 0.058
	U	1313,880						
90% *β* + 10% *γ*	C	408,605	1159,158	135,081	0.919 ± 0.050	0.100 ± 0.004	0.947 ± 0.161	0.125 ± 0.021
	U	1294,239						

All uncertainties correspond to two standard deviations; *C* covered, *U* uncovered

^*^$\stackrel{-}{{N}_{\beta }},\stackrel{-}{{N}_{\gamma }}$ were calculated according to Eqs. 1 and 2

^**^Doses were calculated with Eqs. 3 and 4. Dose uncertainties were 17% according to the procedures at Central Laboratory for Radiological Protection for the range of routine readouts (0.1 mSv—1 Sv). The uncertainty levels may be overestimated occasionally (estimated based on results from a batch of pellets)

**Fig. 7:**
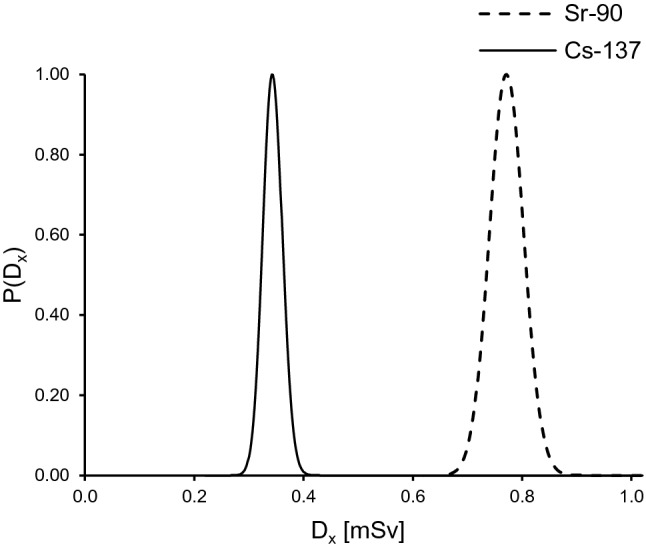
The posterior probability distributions of doses obtained by the Bayesian method for option 1 from Table 1, for 70% beta and 30% gamma radiation

The results of the Bayesian method take the form of probability distributions (see example in Fig. 7). Doses ${D}_{x}$ for the distributions given in Eqs. 15–17 were obtained with the use of Eq. 18 and are presented in Table 4.

**Table 4:** Results of Bayesian method assessment of doses from mixed beta-gamma radiation fields for the three options specified in Table 1

% *β* + % *γ*	Option 1		Option 2		Option 3	
	${D}_{\beta }[mSv]$	${D}_{\gamma }[mSv]$	${D}_{\beta }[mSv]$	${D}_{\gamma }[mSv]$	${D}_{\beta }[mSv]$	${D}_{\gamma }[mSv]$
10% *β* + 90% *γ*	0.122 ± 0.011	0.904 ± 0.042	0.122 ± 0.07	0.902 ± 0.032	0.122 ± 0.012	0.904 ± 0.033
30% *β* + 70% *γ*	0.336 ± 0.018	0.714 ± 0.033	0.336 ± 0.019	0.714 ± 0.024	0.336 ± 0.021	0.714 ± 0.027
50% *β* + 50% *γ*	0.528 ± 0.025	0.512 ± 0.025	0.528 ± 0.029	0.510 ± 0.017	0.528 ± 0.031	0.512 ± 0.021
70% *β* + 30% *γ*	0.772 ± 0.032	0.342 ± 0.018	0.772 ± 0.040	0.344 ± 0.011	0.772 ± 0.041	0.344 ± 0.017
90% *β* + 10% *γ*	0.946 ± 0.039	0.126 ± 0.013	0.946 ± 0.050	0.126 ± 0.004	0.946 ± 0.051	0.126 ± 0.013

All uncertainties are based on a doubled Cramér-Rao assessment of uncertainty (95% HDI, highest density interval)

As one can see, for precisely known expected values of *θ* and *k*_*x*_, the results of the Bayesian dose assessment for the three considered options (Table 1) coincide with the classical one as well as the reference values.

The Monte Carlo method provided very accurate results (Table 5), which are fully consistent with those obtained with the classical and Bayesian approaches (Tables 3 and 4). The main difference relates to the uncertainties which have the same order of magnitude for all methods (Tables 3, 4 and 5).

**Table 5:** Results of Monte Carlo method assessment of mixed beta-gamma doses for three options from Table 1 for 100,000 iterations

% *β* + % *γ*	Option 1		Option 2		Option 3	
	${D}_{\beta }[mSv]$	${D}_{\gamma }[mSv]$	${D}_{\beta }[mSv]$	${D}_{\beta }[mSv]$	${D}_{\gamma }[mSv]$	${D}_{\beta }[mSv]$
10% *β* + 90% *γ*	0.122 ± 0.001	0.904 ± 0.002	0.122 ± 0.008	0.904 ± 0.039	0.122 ± 0.008	0.903 ± 0.039
30% *β* + 70% *γ*	0.336 ± 0.001	0.715 ± 0.001	0.336 ± 0.023	0.715 ± 0.031	0.336 ± 0.023	0.715 ± 0.031
50% *β* + 50% *γ*	0.529 ± 0.001	0.511 ± 0.001	0.528 ± 0.036	0.511 ± 0.022	0.529 ± 0.036	0.511 ± 0.022
70% *β* + 30% *γ*	0.772 ± 0.001	0.343 ± 0.001	0.772 ± 0.053	0.343 ± 0.015	0.772 ± 0.053	0.343 ± 0.015
90% *β* + 10% *γ*	0.947 ± 0.002	0.125 ± 0.001	0.947 ± 0.065	0.125 ± 0.005	0.947 ± 0.065	0.126 ± 0.005

All uncertainties correspond to two standard deviations

## Conclusion

In the present paper, the possibilities of Bayesian statistics, enhanced by the Monte Carlo methods, were examined as a tool in thermoluminescent mixed beta-gamma radiation dosimetry. Three hypothetical situations were considered: either where full information of beam composition and calibration factors was available, or where one or two of the above parameters were unknown (i.e., were given by probability distributions only). To check the correctness of the results obtained by the proposed methods and validate them, Gaussian distributions of prior and likelihood functions with known expected values were considered.

In each of the five considered beam compositions, similar values for ${D}_{\gamma }$ *and* ${D}_{\beta }$ doses were obtained for all presented methods, which confirmed the usability of Bayesian statistics and a Monte Carlo method as alternative methods to the classical approach. The results are therefore very promising, especially regarding situations where dose estimation is beyond the possibilities of the classical method. The described Bayesian and Monte Carlo approaches may prove to be helpful for example in situations where radiation energies are outside the calibration framework, where calibration factors are not known, or where the beam composition is difficult to determine, especially in the case of accidental exposures. It may also be helpful in situations where only one TLD pellet, for example for the lens of the eye lens or for extremities, is available. However, these are just theoretical possibilities which require further work and verification. So far, irradiations of extremity dosimeters in a mixed field have already been performed by the authors, but this requires further calculations and developments.

The Bayesian statistics and Monte Carlo method seem to be good alternatives to the currently used classical approach, but they are much more complicated in comparison with the simpler classical approach. Specifically, they require the application of advanced mathematical calculations and implementation of algorithms in a chosen programming language. At the moment, to the best of the authors’ knowledge, there are no studies that would solve the problem of unknown *θ* or *k*_*x*_ parameters for dose estimation, as well as those that would use the Bayesian or Monte Carlo approaches in beta-gamma mixed fields. So far, doses are assessed directly, most often using different types of passive dosimeters such asTLDs and optically stimulated luminescence dosimeters (Delgado 2000; Chase and Hirning 2008; Ciupek et al. 2014; Carinou et al. 2015; Brame and Groer 2003), scintillators or ionization chambers (Swinth and Sisk 1991).

In the case of dose assessment in mixed radiation fields, Bayesian statistics and the Monte Carlo method were, so far, only applied for mixed neutron-gamma radiation fields including the use of biological dosimetry methods. Thus, the methodologies proposed here are considered original and innovative in the field of TL dosimetry and, even more, their usefulness was demonstrated and confirmed. It is concluded that they can be successfully utilized as tools supporting dose assessment in thermoluminescent dosimetry in mixed beta-gamma radiation fields as well as in other types of mixed radiation fields.

The present study concerned the validation of the proposed methods and demonstrated the actual possibilities of Bayesian and Monte Carlo methods. The proposed methods are still under development by the authors. Irradiations with other radiation sources are already underway. It is also planned to theoretically determine calibration factors for radiation sources that are not available at CLOR, such as ^14^C and others. The next step will also be to use extremity and passive eye lens dosimeters.
